# Regular Leisure-Time Physical Activity is Effective in Boosting Neurotrophic Factors and Alleviating Menopause Symptoms

**DOI:** 10.3390/ijerph17228624

**Published:** 2020-11-20

**Authors:** Boram Kim, Sunghwun Kang

**Affiliations:** 1 Researcher of Leisure and Recreation, Division of Sport Science, Kangwon National University, Chuncheon 24341, Korea; brh@naver.com; 2 Laboratory of Exercise Physiology, Division of Sport Science, Kangwon National University, Chuncheon 24341, Korea; 3 Interdisciplinary Program in Biohealth-Machinery Convergence Engineering, Kangwon National University, Chuncheon 24341, Korea

**Keywords:** cognitive function, menopause, BDNF, cathepsin B

## Abstract

Background: The study investigated the effects of regular leisure-time physical activity on the parameters of cognitive function (plasma brain-derived neurotrophic factor (BDNF), nerve growth factor (NGF), and cathepsin B) and menopausal symptoms (the climacterium, depression, and cognitive impairment) in obese middle-aged women. Methods: All subjects were middle-aged and obese women (*n* = 52, % body fat > 30%). The participants were divided into premenopausal (PRM) (*n* = 18, age = 47.56 ± 6.11 years) and postmenopausal (POM) (*n* = 34, age = 57.79 ± 5.68 years) groups. The participants completed a survey questionnaire related to depression and the climacterium, as well as cognitive tests. Physical activity was performed for 12 weeks. Blood samples from the forearm vein were analyzed after 12 h of fasting. Blood levels of BDNF, NGF, and cathepsin B were analyzed using an R&D kit. Results: Regular leisure-time physical activity had a positive effect on reducing the percentage of body fat in premenopausal and postmenopausal obese women. In addition, the results of the questionnaire showed that regular exercise had a positive effect on body composition caused by lifestyle change and enhanced psychological stability. The BDNF concentration was significantly lower in postmenopausal than in premenopausal obese women. In addition, regular physical activity significantly increased the cathepsin B and NGF levels in postmenopausal obese women. Conclusions: Continuous leisure-time physical activity improved body composition and neurotrophic factors and alleviated menopausal symptoms in obese Korean women.

## 1. Introduction

Female hormones play a central role in a woman’s life, and natural menopause is experienced by all middle-aged women [1,2]. During this period, women face difficulties in accurately describing the physical, psychosocial, and sexual disturbances and mainly report hot flushes, nervousness, depression, insomnia, and general fatigue [3]. The age at menopause is affected by poor physical activity, lower education levels, hormone-based contraception, smoking, and alcohol intake [4]. Middle-aged women experience psychological changes such as depression, isolation, and loss as well as physical changes due to menopause. Appropriate and continuous physical exercises are essential for maintaining bodily function, a healthy lifestyle, and feelings of psychological satisfaction in middle-aged women [5]. In particular, participation in physical exercise has been found to alleviate depression and anxiety symptoms and prevent physiological and psychological changes related to menopause [6]. Therefore, it is important to promote the physical and psychological health of menopausal women through leisure-time physical activities. Physical activity was proven to be an effective method for reducing menopausal symptoms, decreasing bone loss, and increasing muscle strength in menopausal women [7,8,9]. Physical exercise reduces the risk of cardiovascular mortality in postmenopausal (POM) women and is recommended for prevention. Physical exercise has a dose-dependent benefit, and the level of physical fitness has been shown to have an inverse relationship with mortality [10].

Although evidence suggests the benefit of physical activity in combating menopausal symptoms, reports have shown inconsistencies regarding the type or intensity of the exercise and the severity of menopausal symptoms [11,12,13]. Insufficient levels of physical activity have been found to decrease the plasma concentration of cognitive factors and exacerbate menopausal symptoms. Nevertheless, the relationship between regular physical activity, cognitive factors (plasma nerve growth factor (NGF), brain-derived neurotrophic factor (BDNF), and cathepsin B levels), and psychological parameters (the climacterium, depression, and cognition) has not yet been investigated. Thus, this study investigated the impact of regular leisure-time physical activity on cognitive function (plasma BDNF, NGF, and cathepsin B) and menopausal parameters (the climacterium, depression, and cognitive tests) in obese, middle-aged women.

## 2. Methods

### 2.1. Participants

In the present study, the participants included middle-aged obese women (*n* = 52, % body fat > 30%). They were divided into premenopausal (PRM) (*n* = 18, age = 47.56 ± 6.11 years) and POM (*n* = 34, age = 57.79 ± 5.68 years) groups. To be included in the present study, the participants had to (1) be postmenopausal (absence of a menstrual cycle for at least three months) on the date of the assessment; (2) not be receiving hormone replacement treatment; (3) not be using drugs such as beta-blockers and statins; and (4) not have metabolic disease, regular exercise, and diet. The sample size of the subjects was calculated using ANOVA with a large effect size of 0.90, a significance level of 0.05, and a power of 0.80 (G*power 3.1.2). The calculated sample size per group was 15. Prior to participation, all participants provided written informed consent in compliance with the appropriate institutional review board at the Kangwon National University (IRB number: 2016-04-009-002). The participants were also informed of the general study procedures prior to data collection. The general characteristics of study participants are shown in Table 1.

### 2.2. Questionnaire Test

Depression was assessed using the Center for Epidemiologic Studies Depression Scale (CES-D), which was developed by Radloff [14] and used by Park [15] and other studies. The scale contains 20 items that measure depressive symptoms using a 4-point scale, scored as “rarely or not some of the time (less than 1 day)” (0), “some or limited time (1–2 days)” (1), “occasionally or a moderate amount of time (3–4 days)” (2), and “most or all of the time (5–7 days)” (3). Specifically, higher total scores implied a higher degree of depression. Examples of the questionnaire items included: “I concentrate on what I am doing” and “I am patient with others”. In the study of Park [15], the scale showed a Cronbach’s alpha of 0.89. The alpha coefficient was 0.86 in this study.

The Attention Function Index developed by Cimprich, Visovatti, and Ronis [16] was used to measure cognitive function, which was used to assess the ability to carry on without forgetting things while maintaining the usual level of performance in daily life [17]. The visual analog scale displayed in the target area, measuring 0–100 mm in length, was comprised of 13 questions. However, to facilitate its application and response in middle-aged Korean women, the scale was converted to a 5-point Likert scale ranging from 1 (“not at all”, indicative of low cognitive function) to 5 (“extremely well”, indicative of high cognitive function). The participants were asked to report the item that best described the function associated with a specific activity. For example, for the items “keeping your mind on what you are doing” and “being patient with others”, the scale had a Cronbach’s alpha of 0.92 in the study of Cimprich et al. [16]. The alpha coefficient was 0.83 in this study.

Climacteric symptoms were measured with the Korean version of the climacteric symptom index (MENSI) (Jo and Lee, 2001). Sarrel [18] originally developed the MENSI, which was adapted and interpreted for Koreans by Jo and Lee. The scale included 20 items, and the participants were asked to respond regarding the extent of their climacteric symptoms using a “no” (0), “sometimes” (1), or “many” (2). Mild climacteric symptoms were indicated by a score of 10–15 points, moderate symptoms were indicated by a score of 16–29, and severe symptoms were indicated by a score of 30 or more [18]. Examples of the items included in the questionnaire are: “easily excited and angry” and “I feel tired and angry”. The scale had a Cronbach’s alpha of 0.76 in the study of Jo and Lee [19], whereas the alpha coefficient was 0.87 in this study.

### 2.3. Resistance and Aerobic Exercise Program

In our study, the maximal muscle strength (1 RM) was calculated via an indirect method three days before the experiments [20]. First, sufficient warm-up exercises were performed. The subjects were then asked to select the weight that they could lift seven to eight times. A one-time action was defined when it was completed according to a constant rhythm, and formulas were used to define repeated actions based on time and weight [20]. The resistance exercise programs used in the experiments performed by Yeo et al. were modified and adapted to our study (Table 2). Warm-up and cool-down exercises involved 5 min of stretching and 5 min of fast walking on a treadmill at 50% intensity of the maximal heart rate reserve (HRR). Among the resistance exercises, moderate-intensity exercise was defined as circuit training at 55–65% intensity of the 1 RM, repeated 10–12 times in 3 sets. There was a 1-min rest period between the different sets of exercises.

### 2.4. Blood Analysis

Blood samples were drawn from the forearm vein after 12 h of fasting. The blood was centrifuged at 3000 rpm for 10 min, stored frozen at −80 °C, and directly analyzed. Blood levels of BDNF, NGF, and cathepsin B were analyzed using the R&D kit. First, a sample was incubated overnight with 100 µL of capture antibody and washed three times, followed by reaction of the sample and the standard with a detection antibody. Subsequently, the mixture was treated with streptavidin–horseradish peroxidase (HRP) and substrate solution. The reaction was terminated with the kit Stop Solution, and the results were obtained based on an optical density at 450 nm.

### 2.5. Statistical Analysis

The mean and standard error of the data obtained in our study were calculated using the statistical package SPSS for Windows version 22.0. The reliability of the revised instrument was determined by Cronbach’s α. A two-way ANOVA was used to verify the test–retest and intergroup differences in weight. One-way ANOVA was used to verify the temporal variations in blood analysis. Duncan’s post hoc analysis was performed when statistical significance was evident. Statistical significance was set at an α of 0.05.

## 3. Results

The changes in body composition are shown in Table 3. The body weight changes in PRM women at week 0 were significantly higher than those of POM women (*p* < 0.05). However, body fat percentage and BMI were not significantly different. The body fat percentage in PRM women decreased significantly after 12 weeks of exercise compared to the pre-exercise levels (*p* < 0.05). In addition, the levels in PMO obese women decreased significantly between 6 and 12 weeks of exercise compared to pre-exercise levels (*p* < 0.05).

The changes in physical fitness are shown in Table 4. Flexibility in POM women was significantly higher than in PRM women during all exercise periods. In addition, the flexibility in PRM women increased significantly after 12 weeks of exercise compared to the pre-exercise levels (*p* < 0.05). The flexibility in POM women increased significantly after 6 to 12 weeks of exercise compared to the pre-exercise levels (*p* < 0.05).

The changes in depression, cognition, and menopausal symptoms are shown in Table 5. Participation in the exercise program resulted in positive changes in all factors of depression, cognition, and climacteric symptoms without any statistically significant differences. However, there was a significant difference in depression between the groups at 0 and 6 weeks, and the climacterium symptoms were significantly different between the groups at 0, 6, and 12 weeks.

The changes in the neuroplasticity parameters are shown in Table 6. Before exercise, all factors were significantly higher in PRM than in POM women (*p* < 0.05). Blood NGF levels in POM women were increased significantly after 6 and 12 weeks of exercise compared to the pre-exercise levels (*p* < 0.05). The blood BDNF levels in both groups increased significantly after 6 to 12 weeks of exercise compared to the pre-exercise levels (*p* < 0.05). In addition, only POM women showed significantly increased levels of BDNF after 12 weeks of exercise compared to the levels after 6 weeks of exercise (*p* < 0.05). The blood levels of cathepsin B were increased significantly after 12 weeks of exercise compared to the pre-exercise levels in PRM and POM women (*p* < 0.05).

## 4. Discussion

The major findings of this study suggest that POM women experienced depression and climacterium compared to PRM women in obese Korean women. However, the changes in cognitive function were significantly higher in PRM women than in POM women. In addition, regular leisure-time physical activity reduced the climacterium and positively changed the levels of cognitive function. Our findings suggest that psychological depression and menopausal symptoms were clinically stable because regular leisure-time physical activity improved physical fitness and changed the parameters of cognitive function in the blood.

In general, sedentary behavior is recognized as one of the most powerful risk factors for metabolic diseases [21,22]. It is also known that a lack of physical activity is independently associated with a worse immunometabolic profile [23,24]; when this behavior was accompanied by obesity, the risk of death was found to increase 7.5-fold [21]. However, independent of sex and age, moderate activity reduced the chance of death in overweight and obese subjects by 26% and 27%, respectively [21]. The period from perimenopause to POM status not only involves hormonal and metabolic changes but is also associated with changes in body composition, including an increase in body fat and weight gain [25]. However, the relationship between weight, menopause, aging, and hormone levels is poorly understood [25,26]. Several studies assessing the relationship between BMI or body fat and menopausal symptoms yielded inconsistent results [27,28,29].

A study of middle-aged Korean women showed a significant association between obesity and menopausal symptoms [30]. Physical inactivity was found to be an independent risk factor that increased chronic low-grade systemic inflammation and its outcomes [31,32]. In addition, the lack of physical activity is considered a strong risk factor for metabolic disease [21] and was associated with a 7.5-fold higher mortality rate due to a defective immune system and increased obesity [23,24]. Most POM women do not perform regular physical activity. However, regular physical activity maximizes musculoskeletal and cardiovascular performance in menopausal women [33]. The results of the present study showed that regular leisure-time physical activity had a positive effect on reducing the percentage of body fat in PRM and POM obese women. In addition, the questionnaire survey showed that regular exercise was effective against menopausal symptoms due to a positive change in body composition caused by altered lifestyle and enhanced psychological stability. 

Traditionally, most of the studies demonstrating improved cognitive function with exercise were conducted after an acute bout of exercise [34] or chronic aerobic exercise [35]. We determined the impact of the peripheral blood levels of three well-known mediators of brain health attributed to leisure-time physical activity (resistance exercise) on cognitive factors including BDNF, NGF, and cathepsin B [36]. BDNF, a member of the neurotrophin family involved in neuroprotection and neurogenesis, has multiple effects on cell function, including energy metabolism, glucose uptake via mitochondrial biogenesis, and contribution to cellular homeostasis, particularly in the central nervous system [37]. Cathepsin B belongs to a family of lysosomal cysteine proteases and plays an important role in intracellular proteolysis [38]. The upregulation of cathepsin B occurs in premalignant lesions and various pathological conditions, as well as cancer [39,40].

High-intensity aerobic exercise results in a transient increase in serum levels of BDNF in humans [41], which return to baseline within an hour of exercise and may continue to fall below the baseline at 2 and 3 h post-exercise [42]. The effect of chronic aerobic exercise, although less studied, tends to suggest that the resting levels of peripheral blood BDNF are also somewhat increased after a period of endurance training [43]. The results of this study also showed that regular leisure-time physical activity increased BDNF levels. In addition, studies in humans showed decreased plasma levels of BDNF in patients with bipolar disorder, mania, and depression [44,45]. However, not all studies reported differences in the plasma BDNF levels between individuals with depression and the control patients [46]. The relationships between BDNF and depression may be more complex and involve gene-environment interactions [44] or the duration of depression [45]. In this study, the concentration of BDNF was significantly lower in POM obese women than in PRM obese women. However, regular leisure-time physical activity significantly increased the concentration of BDNF in both groups and was more effective in POM obese women. In addition, menopausal symptoms were affected by physical activity. Thus, the decrease in brain plasticity due to menopause leads to psychological stability, and regular physical activity improves physiological brain plasticity.

A recent study reported that treadmill training elevated cathepsin B plasma levels, which may contribute to exercise-induced memory enhancement in humans. Cathepsin B was increased in the plasma after long-term training in mice, rhesus monkeys, and humans [47]. Cathepsin B may mediate the benefits of exercise on brain function via several pathways. Running was found to increase cathepsin B gene expression in the whole hippocampus. It induced hypoxia, which in turn may elevate brain cathepsin B levels [48,49], leading to the clearance of neural debris and adult neurogenesis, a process implicated in memory function [50]. Aerobic exercise is associated with an increase in hippocampal volume. It will be of interest to determine whether cathepsin B levels are correlated with hippocampal gray matter volume [51]. NGF plays an important role in sustaining sympathetic and sensory neurons as well as biological activities, including cell growth [52]. In a recent study, the level of circulating NGF was significantly decreased in fibromyalgia compared with healthy controls. During resistance exercise, the level of circulating NGF was shown to significantly increase in patients with fibromyalgia [53]. However, aerobic ergometry exercises in patients with multiple sclerosis showed no significant difference in the NGF levels of the exercise group [54]. In addition, Bansi et al. conducted a study on three weeks of regular exercise in middle-aged patients with multiple sclerosis, which also failed to significantly alter the resting NGF levels [55]. The results of this study showed a significant increase in the NGF levels of postmenopausal women. It is thought that regular exercise has a positive effect on nerve growth factors in obese patients with menopause.

This study showed that PRM obese women displayed higher levels of positive psychological stability compared to postmenopausal obese women, and regular leisure-time physical activity tended to alleviate menopausal symptoms. In addition, regular physical activity significantly increased cathepsin B and NGF levels in POM obese women.

## 5. Conclusions

Regular leisure-time physical activity reduced the percentage of body fat in PRM and POM obese women and induced positive changes in neurotrophic factors and menopausal symptoms in obese Korean women. Regular physical activity was especially more effective in POM obese women than in PRM obese women, based on all parameters. In addition, based on the results of the questionnaire survey of menopausal symptoms, PRM obese women had higher psychological stability than POM obese women in obese Korean women. In conclusion, continuous leisure-time physical activity improved body composition, boosted neurotrophic factor levels, and alleviated menopausal symptoms.

## Figures and Tables

**Table 1:** Characteristics of participants.

Variable	Contents	*n*	%
Age	40–49	17	32.7
	50–59	21	40.4
	60–69	14	26.9
Education Level	Middle School	3	5.8
	High School	32	61.5
	University	17	32.7
Monthly Household Income	Less than $3000	16	30.8
	$3000 to less than $4000	17	32.7
	$4000 to less than $5000	8	15.4
	More than $5000	11	21.1
Number of Family Members	1	4	7.7
	2	20	38.5
	More than 3	28	53.8
Menopause	Non-menopausal	18	34.6
	Menopause	34	65.4
Total		52	100.0

**Table 2:** Weight-training program.

Exercise	Time	% RM	Intensity
Warm-up	10 (min)	HRR 50%	3 sets 250 kcal rest 60 s
Squat	40 (min)	55–65% RM	
Lunge			
Chest Press			
Vertical Fly			
Lat Pull Down			
Long Pull			
Crunch			
Cool-down	10 (min)	HRR 50%	

**Table 3:** Change of body composition after exercise program.

		0 Weeks	6 Weeks	12 Weeks	Post Hoc
Weight (kg)	PRM	66.7 ± 12.99 *	66.07 ± 11.99	64.92 ± 12.82	
	POM	60.66 ± 6.55	59.80 ± 6.31	59.80 ± 5.71	
% Body Fat (%)	PRM	37.34 ± 6.12	36.05 ± 5.99	34.75 ± 6.14	A > C
	POM	35.26 ± 5.27	33.86 ± 4.87	33.87 ± 4.50	A > B,C
BMI (kg/m^2^)	PRM	26.76 ± 4.57	26.58 ± 4.41	26.20 ± 4.87	
	POM	24.68 ± 2.54	24.30 ± 2.36	24.41 ± 2.26	

Mean ± SE, *p* < 0.05, PRM: premenopausal group, POM: postmenopausal group, BMI: body mass index, * between-group difference, A: 0 weeks, B: 6 weeks, C: 12 weeks.

**Table 4:** Change of basal physical fitness after exercise program.

		0 Weeks	6 Weeks	12 Weeks	Post Hoc
Grip strength, left (kg)	PRM	21.54 ± 1.75	22.48 ± 1.30	22.18 ± 1.51	
	POM	19.30 ± 1.23	21.96 ± 1.04	21.37 ± 0.92	
Grip strength, right (kg)	PRM	22.72 ± 2.28	23.49 ± 1.59	23.16 ± 2.09	
	POM	20.64 ± 1.34	23.04 ± 1.01	22.95 ± 0.90	
Trunk forward flexion (cm)	PRM	12.61 ± 1.98 *	15.87 ± 1.57 *	14.76 ± 1.99 *	
	POM	17.47 ± 1.30	20.26 ± 1.19	19.94 ± 1.33	
Sit-up (Frequency)	PRM	16.46 ± 3.16	18.53 ± 3.40	23.46 ± 3.26	
	POM	10.85 ± 1.90	16.05 ± 2.36	18.05 ± 2.12	
Standing broad jump (cm)	PRM	130.38 ± 7.56	138.00 ± 6.67 *	133.15 ± 7.38	
	POM	114.25 ± 4.89	118.85 ± 3.94	125.55 ± 5.21	
Sidestep (Frequency)	PRM	29.00 ± 1.29	32.61 ± 1.33	34.15 ± 1.48	A < C
	POM	28.65 ± 0.94	32.20 ± 0.64	33.75 ± 0.70	A < B,C

Mean ± SE, *p* < 0.05, PRM: premenopausal group, POM: postmenopausal group, * between-group difference, A: 0 weeks, B: 6 weeks, C: 12 weeks.

**Table 5:** Changes in depression, perception, and the climacterium after exercise program.

		0 Weeks	6 Weeks	12 Weeks	Post Hoc
Depression	PRM	11.07 ± 1.42 *	10.15 ± 1.56 *	10.23 ± 1.50	
	POM	15.05 ± 1.71	14.30 ± 1.89	13.65 ± 1.40	
Perception	PRM	3.51 ± 0.64	3.62 ± 0.63	3.55 ± 0.43	
	POM	3.44 ± 0.38	3.44 ± 0.39	3.50 ± 0.44	
Climacterium	PRM	7.46 ± 1.55 *	7.69 ± 1.72 *	6.15 ± 1.78 *	
	POM	12.05 ± 1.64	12.15 ± 1.65	11.05 ± 1.36	

Mean ± SE, *p* < 0.05, PRM: premenopausal group, POM: postmenopausal group, * between-group difference.

**Table 6:** Changes in neuroplasticity factors after exercise program.

		0 Weeks	6 Weeks	12 Weeks	Post Hoc
NGF (pg/dL)	PRM	200.43 ± 79.73 *	207.26 ± 21.28	206.18 ± 12.16	
	POM	190.23 ± 5.59	199.83 ± 12.46	206.05 ± 12.29	A < B,C
BDNF (pg/dL)	PRM	294.99 ± 50.97 *	349.11 ± 50.96	378.47 ± 48.63	A < B,C
	POM	206.00 ± 27.74	242.38 ± 36.41	280.68 ± 44.48	A < B,C B < C
Cathepsin B (ng/dL)	PRM	214.11 ± 87.77 *	242.06 ± 79.73	262.35 ± 81.37	A < C
	POM	181.87 ± 55.59	211.89 ± 65.36	225.87 ± 67.08	A < C

Mean ± SE, *p* < 0.05, PRM: premenopausal group, POM: postmenopausal group, * between-group difference, A: 0 weeks, B: 6 weeks, C: 12 weeks.
